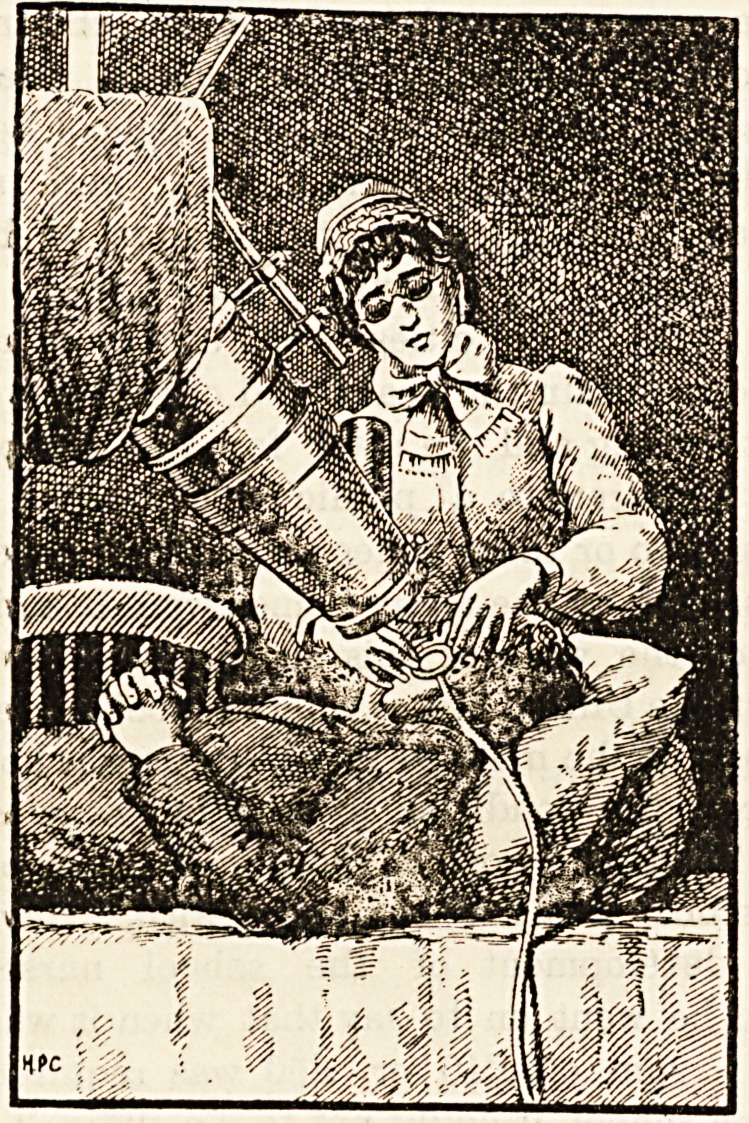# The Hospital. Nursing Section

**Published:** 1903-05-30

**Authors:** 


### The Hospital.

### Nursing Section.

Contributions for this Section of "The Hospital" should be addressed to the Editor, "The Hospital" Nursing Section, 28 & 29 Southampton Street, Strand, London, W.C.

No. 870.—Vol. XXXIV. *SATURDAY, MAY* 30, 1903.

### Notes on News from the Nursing World.

### THE QUEEN'S GIFT TO HER NURSES.

It was intimated in the daily papers on Tuesday that the Queen has given £1,000 towards the provision of a new habitation for the Queen Victoria Jubilee Institute of Nurses, and that her Majesty's gift was made with an entire absence of ostentation and formality, being received in the form of a cheque enclosed in an envelope with the following autograph message written on the envelope:—"£1,000 for new offices for the Queen's Nurses now at St. Katherine's.—Alexandra." Since then it has been announced that the statements with regard to the future arrangements for the offices of the Institute were not authorised by the Council.

### THE WOMEN'S MEMORIAL TO QUEEN VICTORIA.

Not only the ladies who had the honour of being received at Buckingham Palace on Thursday, last week, but also the thousands of the fair sex throughout the country who worked so hard in order to render the Women's Memorial to Queen Victoria a fitting tribute, must have felt repaid for their energy by the gracious manner in which the King and Queen acknowledged the outcome of their efforts. When their Majesties had received from Lady Londonderry, as president of the executive committee of the fund, a draft for £66,050, and one from Lady Cadogan on behalf of Ireland for £6,000, the Duchess of Buccleuch having previously presented the sum of £12,000 on behalf of Scotland, for the purpose of helping to endow the Queen's Jubilee Institute for District Nurses, the King said: "I congratulate you on the success of your kindly labours, and I am very glad that so large a sum has been contributed by so many people to so worthy an object as the Queen's Nurses. It is an additional pleasure to the Queen and myself that this sum should have been collected as a memorial to my beloved mother." The Queen, as patron of the Queen's Nurses, also expressed her thanks. As it was stated at Buckingham Palace and at the winding-up meeting of the executive on Friday, when the report of the committee was formally adopted, there were four millions of subscribers, nine-tenths of the total raised being in small sums ranging from a penny upwards. This is the most notable, and the most satisfactory feature of the memorial, and affords yet another remarkable proof of the importance of collecting pennies. It is only fair to add that Mr. Harold Boulton, the honorary treasurer of the Queen's Jubilee Institute, and all the officers of the organisation, are thoroughly entitled to the cordial vote of thanks which the committee accorded them. Congratulations are due to them no less than to the organisations of ladies upon the results of their labours.

### THE WORK OF THE QUEEN'S NURSES.

The submission to the Queen of the annual report of Queen Victoria's Jubilee Institute for Nurses by the council was appropriately made in the same week as the presentation of the substantial sum collected for the further endowment of the charity. One of the most interesting parts of the report has reference to the popularity of the movement among the mining population. In several places, as at Treorchy in South Wales, Cefn in North Wales, Willington in Durham, and Radcliffe Colliery in Northumberland, the miners have themselves taken steps to secure the services of a Queen's nurse. At Cefn the local Association is almost entirely kept going by small contributions from 6d. to 5s., and at Radcliffe the 1½d. a fortnight given by the miners suffices, with one subscription of 5 guineas, to support a nurse. The number of nursing associations in affiliation with the Institute continues to increase, and on December 31st last was 563, as compared with 521 at the end of 1901. The total number of Queen's nurses on the roll at the same date was 950, as against 858, and in addition to these there were also at work 96 nurses who became Queen's nurses on January 1st, 1903. Since 1902, 41 former Queen's nurses have rejoined the Institute. In the supplementary report of the council and committee of the Queen's Commemoration Fund, it is stated that less difficulty was experienced last year than previously in finding suitable nurses for training, and that the very large increase in the number of applications in England and Wales has been a very encouraging feature. General interest in the Queen's Institute and its work has also, it is mentioned, been aroused and increased to a very great degree by the efforts on behalf of the Women's Memorial Fund, and in many places the wish to have a Queen's nurse has been the immediate result. Unfortunately, there are still insufficient nurses to meet the demand, and the council are thus brought face to face with the alternative of either substantially increasing their income, or reducing the area of their work at a time when, more than ever before, it is needed and valued throughout the country. Allowing for the income from the Women's Memorial Fund, a further sum of £1,500 a year is required to meet the existing deficit, and provision for the normal increase of expenditure at the rate of £700 a year is also needed. Moreover, to enable the council to attract the most suitable nurses to district work, and to retain them, funds are necessary in order to improve the position and prospects of the workers. It is therefore quite clear that the friends of the Institute must redouble, rather than relax, their efforts on its behalf if the beneficent movement is to be extended without trenching very much more on the available capital of the Commemoration Fund.

### THE LATE DR. SOLOMON SMITH.

AT a recent meeting of the Executive Committee of the Workhouse Nursing Association, the following resolution was moved by Miss Wilson, seconded by the Hon. Mrs. J. G. Talbot, and unanimously carried:—"That this committee record with deep regret the loss sustained by the death of Dr. Solomon C. Smith, whose keen knowledge of and interest in questions affecting Poor Law nursing rendered him a valuable adviser and an able fellow-worker. Dr. Smith's place is not one that can be easily filled, and the cause of the efficient nursing of the sick poor, which presents many difficulties, has lost a zealous worker, and a true friend."

### BRITISH LYING-IN HOSPITAL.

ALTHOUGH still in the hands of the workmen, the nurses' home at the British Lying-in Hospital, which the Princess of Wales will open next month, is practically finished, some of the rooms being indeed already in use. The new dining-room is situated in the basement, and has a dome-shaped roof. Greenish blue and white is the main scheme of colour everywhere, and it is employed with very pleasing results. There are capacious linen and other cupboards, and there is a very neat arrangement of pigeon-holes for the nurses' boots and shoes, each having a number corresponding to that of the bedrooms. White tiles are used in the offices, and a new invention, consisting of variously-coloured slabs made of broken glass and cement, lines the staircase, and adds to the general effect. The separate sitting-rooms for nurses and pupils are on the entrance floor, and are comfortably furnished with lounges, and lighted with electric light, while pretty green linen curtains hang in the windows. The 20 bedrooms are on four floors, and are approached by a steel and concrete staircase covered with teak, while a judicious use of white paint and curved lines make the hall and passages pleasing to the eye. The bed-sitting-room occupied by the assistant matron has a Berliner telephone fixed in case of emergency. As already announced, the opening ceremony will take place at 3.30 on Monday, June 8th prox. Her Royal Highness will be received at the entrance to the home, in Betterton Street, and conducted over the new building by the matron, Miss Gertrude Knott. The Princess has consented to receive purses containing not less than £5, to be devoted towards wiping off the debt of £7,000.

### HOSPITAL FOR EPILEPSY AND PARALYSIS, MAIDA VALE.

THE nurses' rooms at the Hospital for Epilepsy and Paralysis are practically ready for occupation, but owing to unfortunate circumstances in connection with the flooring of the passages throughout the building, there will probably be some delay before they can be taken possession of by the prospective staff. The bedrooms and sitting-room are on the top floor of the new building facing Maida Vale, and are pretty rooms with plenty of light and air. Three of the bedrooms have Teale fireplaces, the remaining five being warmed by radiators in the passages. In addition to large windows, each door is fitted with a sliding ventilator. The furniture is useful and solid, and the hanging cupboards are supplied with revolving hooks. Primrose walls relieved by a white frieze, with a narrow green picture-hook line, distinguish the nurses' rooms, which are attractive and comfortable-looking. The matron's apartments divide the nurses' quarters from those of the domestic staff, and each set has separate bathroom and lavatory arrangements. The dining-room is on the basement, with colouring of primrose and blue, blue tiled fireplace, blue linen curtains and green stained sideboard. The opening ceremony is to take place on June 13th, when H.R.H. Princess Louise, the Duke of Argyll, and others are expected to be present.

### THE NURSE IN PRISON.

WITH reference to the article under this heading which appeared in the *Nursing Section* of 25th ultimo, page 41, Dr. Donkin is very anxious that it should be clearly understood that he is not ready to receive applications from nurses for appointments. Nurses should understand clearly that there are practically no special appointments, as such, for nurses in the prison service. Nurses are selected from among the general body of prison officers according to their special fitness. All female officers are required to enter the prison service on the same basis, and to undergo the same general training. The main point of interest to nurses is, however, that candidates for the office of assistant warder who are qualified nurses are regarded as desirable. A statement as to prison warders' salaries will be found on page 81 of the *Nursing Section* of THE HOSPITAL for May 9th.

### THE ANSWER TO A BITTER CRY FROM BRISTOL.

THE bitter cry from Bristol, to which we referred last week, has not fallen on deaf ears. The lady superintendent of the Bristol District Nurses' Home informs us that on May 22 the committee received a telegram from Mr. George White, dated the Elysée Palace Hotel, Paris, asking them to be kind enough to immediately replace a nurse in the Moorfields region, and stating "I will pay the £70 this year." Mr. White, who is the managing director of the Bristol Tramways and Carriage Company, intimated that the committee would thus have ample time for getting a permanent subscription list, which, he added, "You will easily do in Bristol." With such an admirable example before them, we cannot doubt that the citizens of Bristol will justify Mr. White's forecast, and take care that there is never again a question of withdrawing a nurse from the homes where she is most wanted.

### AN ARMY SISTERS BROKEN ENGAGEMENT.

A QUESTION of interest to nurses already resident in or likely to proceed to South Africa was argued in the Court of Appeal before Lord Justices Stirling and Mathew on Monday. Miss Jennie Crosbey, who was trained at the Middlesex Hospital and had been a nursing sister in the Army Nursing Service Reserve for nearly three years, became engaged whilst she was at Wakerstroom, South Africa, to Captain Reginald Drake, of the 2nd North Staffordshire Regiment. This was in May, 1902. At the request of her fiancée, Miss Crosbey, in anticipation of her marriage, severed her connection with the Nurses' Co-operation, where she had been employed for five years. In February, 1903, Captain Drake wrote from Piet Retief that for many reasons he felt he must ask Miss Crosbey to release him from their engagement. This was partly because of his financial position, and he expressed a hope that she would allow his parents to compensate her for any expenses she had incurred in getting ready for their proposed marriage. Captain Drake's father also repeated this hope. Miss Crosbey's counsel asked for leave to appeal from an order of Mr. Justice Phillimore refusing to allow a writ to be served on the captain in South Africa for alleged breach of promise to marry. But both the Judges upheld the decision, maintaining that the contract to marry was made in South Africa, and broken in South Africa, when Captain Drake posted the letter to Miss Crosbey. Consequently, leave could not be given to serve a writ out of the jurisdiction, and Miss Crosbey could only sue the defendant by going out to South Africa and entering proceedings in the courts there.

### THE NURSING SCANDAL AT GRANARD INFIRMARY.

The Granard Board of Guardians have been severely censured by the Irish Local Government Board. This is the result of a long and careful inquiry into the provision made, or not made, by the Guardians for the care of the sick and infirm under their charge. The evidence given revealed, in the opinion of the Local Government Board, a shocking condition of things, and but for the admissions of the chairman and other Guardians which appeared to show that they are at last alive to the necessity for making improvements, their default would have involved dissolution. One of their gravest errors was that "when the medical officer requisitioned a nurse he was called upon by them to resign because he would not permit the wardmaid to do the nursing duties." This action the Local Government Board describe as "a most improper attempt to intimidate an officer in the conscientious discharge of his professional duty." On the other hand they affirm that the evidence shows that "the workhouse master has been remiss, careless, and inefficient in the discharge of his duty," and unless their inspector is able, on another occasion, to report less unfavourably of him, he will not be permitted to remain in office. We observe that some of the Granard Guardians propose next week to take into consideration "what relations should in future exist between the Board and the medical officer." There is here a suggestion that an effort will be made to render things unpleasant to the medical officer who has so admirably fulfilled his obligations. The Irish Local Government Board may, however, be relied upon to take care that Dr. Kenny is not hampered any further in his work. Even if he throws up his post in disgust, the days of permitting a wardmaid to perform the functions which belong to a trained nurse are over.

### BRITISH NURSES' ASSOCIATION.

Princess Christian will preside at the annual meeting of the Royal British Nurses' Association to be held at the Imperial Institute, on Saturday, June 6th, at 3.30 p.m. The members will meet afterwards at the Earl's Court Exhibition, and tickets for admission, including tea, can be obtained from the secretary at the offices in 10 Orchard Street, W.

### THE CERTIFICATE OF WORKHOUSE INFIRMARY NURSES.

The Westminster Board of Guardians have decided that in future an independent doctor shall be employed to examine the nurses in the infirmary and sign the certificates. Mr. Brass, the originator of the change, stated at the meeting of the Board at which the question was discussed, that a case had recently come to his notice of a nurse being refused a situation in consequence of the certificate being signed by the doctor under whose supervision she was instructed. Independent examinations in Poor Law unions are very useful, but the certificate of a qualified medical superintendent should be accepted as sufficient proof that a nurse had been properly trained. It will next be proposed that the matron under whom a nurse is trained is not a suitable person to sign a certificate.

### THE ASYLUM NURSE IN AMERICA.

A school for trained nurses is about to be established in connection with the State Hospital for the Insane at Trenton, U.S.A. Miss Rachel Bourke, for 13 years head nurse of Cooper Hospital, is to be the first head of the training school, and will take up her new duties on September 1st. In the interval she will spend a couple of months in Europe.

### HEALTH VISITORS FOR POOR DISTRICTS.

By permission of the Duke of Westminster, the successful candidates at the National Health Society's examinations received their diplomas and certificates at Grosvenor House, on Saturday afternoon. The presentations were made by Princess Christian, and there was a large attendance, among others present being the Baroness Burdett Coutts, Sir Dyce and Lady Duckworth, Sir William and Lady Church, and Sir James Crichton Browne. The Earl of Derby occupied the chair. The need for "Health Visitors" among the poor of London was indicated by the Bishop of Stepney, who said that some method of co-ordinating the public officers of health, those in personal touch with the poor, and the poor themselves, was wanted. This was supplied in Stepney by a voluntary association of persons interested in the matter, and a lady would shortly be appointed whose duties would be somewhat similar to those of the health visitors in Glasgow, Manchester, and other towns, viz., to visit the homes of the people and inculcate personal and domestic hygiene, sanitation, and the care of infants. One of the recipients of the full diploma of the Society was Miss Buckley Williams, a nurse trained at the London Hospital; and another, Miss Marion Spencer, had received six months' hospital training.

### SHORT ITEMS.

At the examination recently held at the Cancer Hospital, Fulham Road, London, on the termination of the lectures given by the medical staff during the winter session, the first three places were secured by Nurses H. Hawkins, M. Watts, and A. Dow, to whom the three prizes offered by the Committee of Management will be awarded.—Miss M. Griffith, the new matron of the Royal Mineral Water Hospital, Bath, was trained at the Bristol General Hospital, as well as the Manchester Hospital for Sick Children, Pendlebury.

### The Nursing Outlook.

"From magnanimity, all fear above;
From nobler recompense, above applause,
Which owes to man's short outlook all its charm."

### THE TRAINED MASSEUSE.

The interesting particulars given in an interview with Miss Vernet of the National Hospital for the Paralysed and Epileptic, which we publish to-day, suggest that it may be well to look forward into the future of the massage movement. Massage is one of the oldest professions in the world—we find mention of it in early Jewish and Egyptian days, and Epictetus, the slave philosopher, describes as one of the delights of the rich—"so that when you have stripped yourself in the bath, and stretched yourself out as though you were crucified, you may be rubbed to and fro, and the rubber standing by may say: 'Turn him round, give me his side, take hold of his head, let me have his shoulder'; and then when you go home you may shout, 'Is no one bringing me anything to eat?'" And it was as "rubbers" that the work was carried on down many centuries, till Germany and Sweden took to more elaborate manipulations and exercises under medical direction, and the terms "massage," "masseuse," and "masseur," were introduced, to distinguish the medically-trained manipulator from the old-fashioned rubber. About 15 years ago the work caught on enthusiastically in England; then there was gradual doubt and detraction, and in the autumn of 1894 the medical and daily papers gave publicity to "Massage Scandals," which threw the work into disrepute, and the practice became regarded with suspicion.

But while medical men fought shy of the unknown and untrained masseuse, they became all the more anxious to secure the services of reputable and skilful persons, and gradually massage settled down as a successful adjunct to certain forms of treatment, but was no longer regarded as a panacea for every ill. This re-establishment of the remedy in public opinion was largely due to the steady support of the National Hospital, of Dr. Stretch Dowse, and others, and also to the formation of the "Incorporated Society of Masseuses" in January, 1895, for the purpose of improving, training, and organising an examination for competent masseuses. The Society worked very quietly for a time, but its success, though slow, was sure, and it now holds an examination three times a year, and has about 50 candidates at each examination. It has its meetings and classes at the Nurses' Club, 12 Buckingham Street, Strand, and there the secretary can be seen on Fridays and all details can be obtained. But the Society is very modest, and it has not taken the position it might have done had it possessed more vigorous and courageous leaders. It often seems to us that there is no profession which so lacks "go" and combination as the nursing profession. The vexed questions of the needful natural gifts of a masseuse, of the length of training, of the proper standard of knowledge for gaining a certificate, and so on, have never been laid down, and the result is that the medical man who employs a masseuse to-day has no fixed guarantee of her proficiency unless she has been trained in his own school. The training varies from two years to two weeks. It may include anatomy, physiology, electricity, and general nursing, or it may merely include a few rubbing movements hidden under the high-flown titles of "tapotement," "effleurage," and so on. It has never even been decided whether massage should be taken as an adjunct to general nursing, or whether it should be regarded as a quite distinct profession. It has never been decided as to whether theoretical lectures and practice on a dummy are sufficient, or whether practical work in the wards is necessary before certification.

Mrs. Creighton-Hale—one of the oldest teachers—has chosen the middle path of making her pupils practise on one another. As the present apathy leaves the way open to schemers who are always waiting for opportunities of advertising themselves, would it not be well for the Society of Trained Masseuses to take the field boldly, to secure the co-operation of all training schools, to organise conditions and associations, and to establish the status of the workers? We believe massage to be a branch of the nursing profession; we maintain that it should never be taught except to nurses who have had a general training; and we think that it should only be taught to those whose natural qualifications fit them to become expert. With regard to the first point we contend that the medical man leaves so much to the masseuse that the general training is necessary to guide her: it is not merely a question of massage if you receive orders to give two and a half hours' treatment a day to a case; much depends on the time and movements given. Insomnia needs treating towards evening; exhaustion cases also, if treated very early, simply remain over-tired all day; but constipation needs morning treatment. These questions may not come up in Germany where rubbing is given in clinics with doctors looking on, but it is different here. With respect to length of training, we consider that three months should be the minimum, and we are of opinion that the exercises should be practised on the human body. But it is not for us to dictate in these matters: it is for the masseuses themselves to boldly formulate a policy and follow it.

### Lectures on Ophthalmic Nursing.

By A. S. COBBLEDICK, M.D., B.S.Lond., Senior Clinical Assistant Royal Eye Hospital, late House-Surgeon and Registrar, Royal Eye Hospital.

### LECTURE XI. — CONJUNCTIVITIS — ACUTE, SIMPLE —SYMPTOMS, DIAGNOSIS, PROGNOSIS, AND TREATMENT.

CONJUNCTIVITIS, or inflammation of the conjunctiva, is one of the commonest affections met with in ophthalmic practice; this is no doubt partly due to the fact that a very sensitive membrane is much exposed to irritation from without—in spite of the protective action of the eyelids—but also to the fact that most inflammatory affections of the conjunctiva are contagious; thus it is that a whole family may be under treatment, in different stages of the disease, through neglecting to isolate the member first affected.

All degrees of intensity of inflammation are met with, varying from slight discomfort and pricking of the eyes to a profuse yellow discharge with œdema of the conjunctiva and lids, and possibly very serious complications, endangering the sight and even the eye itself.

*Simple Acute Conjunctivitis* is caused by contagion, *e.g.*, when an infected person soils a towel which is subsequently used by other members of the same family; epidemics of this trouble are met with especially in dry windy weather, with much dust in the air; sitting in a cold draught is not uncommonly an exciting cause; it frequently follows measles, scarlet fever, and diphtheria. It is now generally supposed that this disease is bacillary, and is caused by a long rod-shaped bacillus which grows readily in blood serum.

*Symptoms.*—The onset may be gradual or sudden; it is sometimes so sudden that the patient is under the impression that a foreign body has blown in the eye. In such a case there is considerable pain of a pricking, burning character, much lachrymation, and not unfrequently photophobia (inability to keep the eye open in a strong light).

In the course of a few hours a sticky discharge makes its appearance along the edges of the lids and at the external and internal canthi. After a night's rest it is found in the morning that the lids are firmly gummed together and cannot be opened until they have been bathed with warm lotion; this is a valuable symptom when there is some doubt as to the degree of conjunctivitis present.

On drawing down the lower or everting the upper lid of the affected eye the conjunctiva covering them is seen to be red and swollen; in severe cases, especially if they have been left untreated for some time, small red elevations—swollen papillæ—are present. The conjunctiva covering the "white of the eye" is not so frequently affected, and in slight cases there is no departure from the normal.

In severe cases the conjunctival blood-vessels are enlarged and small grey patches may be seen at the edge of the cornea; if one or more of these coalesce a crescentic marginal corneal ulcer results. As the ulcer begins to heal its blood supply becomes greater and a leash of conjunctival vessels stands out very conspicuously.

*The Prognosis* in all these cases is good; even in a severe case the cornea does not become affected excepting at its margin, and with appropriate treatment these marginal ulcers need cause no great anxiety.

*Diagnosis.* — Slight cases must be distinguished from irritative conditions caused by eye strain, the presence of foreign bodies, and the use of irritant—home-made—applications, *e.g.*, tea-leaf poultices, hot milk, etc.

Foreign bodies in particular may set up great injection of the conjunctival vessels, and if it is a minute particle of sharp flint a very careful search is necessary on the cornea as well as on the lids. The point of importance is, however, that in none of these cases of simple irritation is there a gummy discharge. It is possible, however, for a foreign body to carry some infection with it into the conjunctival sac and set up some conjunctivitis. Engorgement of the conjunctival vessels must also be distinguished from the redness caused by injection of the vessels in the sclerotic—the small anterior ciliary vessels. The latter vessels are enlarged in inflammation of the ciliary body and of the iris, so there is a possibility of mistaking cyclitis and iritis for conjunctivitis. The deep vessels are smaller and form a salmon-pink coloured zone extending around the margin of the cornea for a quarter of an inch. Unlike the conjunctival vessels, they do not move with the conjunctiva on manipulating it with the lower lid. Unless the examination of the eye is careful, a small transparent corneal ulcer may be mistaken for simple acute conjunctivitis.

In all these cases—simple irritations, iritis, cyclitis, and corneal ulcerations—the important guide, which serves to distinguish them from conjunctivitis, is the absence of discharge.

*Treatment.*—In the early stage, where there is much burning and pricking, an iced boracic pad, frequently changed, gives great relief; later, it is necessary to irrigate the conjunctival sac with warm boracic acid lotion (gr.v. to gr.x. ad. ℥i.). In a severe case the irrigation should be carried out every two hours; slighter cases require attention less frequently—three or four times a day.

At night, if the edges of the lids are allowed to adhere retention of secretion in the sac takes place and recovery is impeded, so it is important to order Unq. Ac. Borici. (4 per cent), to be applied at bed-time.

As a rule no further treatment is necessary, and a cure should take place in a week or ten days' time; if the marginal ulceration is extensive, it is advisable to instil weak atropine drops, gr. i.–ii. ad. ℥i.

*Instructions to the Patient.*—Almost without exception, patients when instructed to use an eye lotion, bend their heads over a bowl, close thelr lids and bathe the outside of the lids, a procedure which readily explains many cases of slow recovery. It is therefore most important to explain that the lotion must be applied to the inside of the lids, and that it should be carried out by a second person; when time admits a demonstration should be given.

*How to Bathe an Eye.*—Prepare the lotion in a basin rendered aseptic by cleansing with boiling water. Place in the lotion small pledgets of sterilised absorbent cotton wool; a receiver or large piece of absorbent wool is placed in contact with the side of the face. Stand behind the patient, who should be seated in a low-backed chair, draw the patient's head backwards, so that the gaze is directed upwards and fix it firmly against the chest; in this manner the patient, however nervous, can be readily controlled. In keeping the lids open, be careful to press the skin against the bony orbital margin and not against the eyeball, the conjunctival sac can then be thoroughly irrigated.

*Follicular Conjunctivitis.*—In causation and symptoms this very much resembles the form already described. It is, however, a very much more chronic disease, and may persist for months.

Its chief characteristic is the presence on the palpebral conjunctiva, especially of the lower lid, of small, round, pinkish elevations about the size of a pin's head; these must not be confused with the more translucent granules seen in granular ophthalmia, to be hereafter described. The ocular conjunctiva is not affected.

*Treatment.*—This must be of a more stimulating character. A lotion of chloride of zinc gr. ii. ad. ℥i., alone or combined with boracic acid lotion 4 per cent. is useful, or drops of sulphate of copper gr. ii. ad. ℥i. Attention must also be given to the general health, and any co-existing error of refraction must be corrected.

### London School Nurses' Society.

By permission of Lady Windsor a meeting was held on behalf of the London School Nurses' Society on Tuesday last at 54 Mount Street. Lady Windsor presided and requested the Hon. E. Lyulph Stanley (chairman of the Executive Committee), to address the meeting.

Mr. Stanley stated that the society had been at work for about five or six years and did as much as it possibly could with the very scanty means at its disposal. It was purely voluntary. Its chief object was to get at the poorer children in the schools and attend to their minor ailments, such as sore heads, sore eyes, etc., which were far more prevalent than they should be. A school nurse was not needed in all schools, but in at least one-quarter of the schools in London her presence was most useful. The founders of the society did not contemplate its becoming a permanent institution, but their aim was to show the way that others might follow in their steps. They much desired to be able to increase the salaries of their nurses and thus to secure a better class of nurse.

Lady Jeune said that the prevalence of sore eyes and the bad condition of the children's teeth was noticeable to all who visited the schools. Much was done for children nowadays, and yet there were vast numbers to whom no help had yet been given. The nurses were very popular with the children, who were quite glad and thankful to be seen to. If anyone desired to do a good work there was no better way than by guaranteeing the salary of a nurse. Great benefit had been found to result from the attendance of the district nurse at schools in the country.

Mr. Evelyn Cecil, M.P., said that he had been connected with the society from the beginning, including the time he was working on the London School Board, and he had always thought that something of this kind was required. He was glad to notice among the objects of the society a special desire to educate and strengthen the parents' responsibility. In large towns, and especially in London, parents were often only too desirous of getting rid of their children and sending them to school as early as possible. It was sometimes urged that the work done by voluntary societies should be paid for out of the rates, but he deprecated this idea. There was far too great a disposition in the present day to consider that the State could take the place of charities. It was a pride and a privilege to help charitable institutions, either by money or by personal service, and he hoped that each year would see a material increase in the funds as well as in the work of the London School Nurses' Society.

Miss Susan Lawrence, hon. treasurer, said that the object of the society was to help some of the most neglected children in London. It was difficult to realise how very low the standard of ordinary necessaries among the poorer children was. She gave an instance of some six-year-old children whose underclothing was found to be firmly sewn on to them, and said that the contrast between the fine school buildings and fittings and the poverty of the little scholars was most striking. The income of the society only reached £180, which provided three or four nurses; if, however, they could reach £500 or £600, they thought that sum would be quite sufficient. The society was entirely voluntary, except for one item which was provided by the generosity of the School Board, who, recognising the good results of the nurses' work, decreed that a kettle should be provided for their use, but took care to add that its cost must not exceed 3s.

Sir Henry Burdett said that he could not agree that this was work that ought to be done entirely by charity. At the present time, however, they had no alternative but to continue it as a charity. We had in this country a system of national education which was free, and in which compulsory attendance at the schools was insisted on. It certainly was a reflection on our legislators that having provided free education, and having compelled the attendance of the children, they omitted to look to the necessary hygienic and sanitary requirements, which would bring the children into an adequate condition for receiving the learning and knowledge their teachers were prepared to instil into them. The only schools where such requirements were attended to were those for the mentally deficient, where by the regulations in force every child was bathed at least three times a week. The result was that although the children attending those schools were drawn from the same class as those at the public schools, there were not to be found among them 90 per cent. with dirty heads. This was a very unpleasant subject, but in this discussion unpleasant subjects must be touched upon. The ears, eyes, and heads of the children rendered them sources of danger to their fellows. He considered that it was high time that the London School Board, instead of squandering money over pianos and such items, should turn their attention to matters of hygiene and not limit their expenditure under that head to a 3s. kettle. In other cities and other countries much was done to maintain the health and cleanliness of the child. It was quite wrong and an utter farce to compel people to send their children to a public school when they might return home two or three times a week in a condition of uncleanliness. Of course many homes were insanitary, but as the result of the work of this society, and it was the same principles in the United States where school nurses were instituted, so soon as the mothers had been taught how to care for the children, the condition of the homes was materially improved. Thus the work of the school nurse benefited not only the school but the commonwealth. Having touched upon the development of the school nurses' work in New York, he went on to say that when it was considered that only a sum of £40 or £50 was required to secure a nurse for a school, it ought not to be difficult to get eight or nine people each to provide a nurse, and each nurse might be known by the donor's name. He thought that strong representations should be made to those in charge of the new Education Act to get them to make it a matter of law that the physical welfare of the children should receive careful attention and that the public schools should be placed in as good condition as those for the mentally deficient. He concluded by moving a vote of thanks to Lady Windsor for presiding and lending her house for the meeting.

The proposal was seconded by Sir Arthur Clay, who differed from Sir Henry Burdett on the point of State aid in this connection.

Lady Windsor in responding made an appeal for help towards the funds of the society.

### "The Hospital" Convalescent Fund.

The Honorary Secretary begs to acknowledge, with thanks, the receipt of 2s. 6d. from Miss Agnes Thomson.

### The Finsen Light Treatment.

BY HONNOR MORTEN.

### II.—THE FINSEN LAMP.

SUNLIGHT having proved itself too unreliable and irregular to be trusted with the cure of the hundreds of lupus cases clamouring for the new treatment, Dr. Finsen invented a powerful electric arc-lamp from which projected four telescopic tubes down which the light was focussed to four couches where the patients could recline. Also he procured coolness by a jacket of water to the telescopes, and a constant stream of cold water through hollow-pressure glasses and thus cut off the heat rays without using the copper solution.

A lamp of this description was presented to the London Hospital by Queen Alexandra and installed on May 29th, 1900. By means of a transformer a 30,000 candle-power was obtained, and the lamp is usually worked at from 50 to 65 ampères. The "electric arc" light is produced by the constant passage of a current of electricity from a powerful battery between poles of carbon; and an ampère is merely the unit of measure of strength of an electric current.

Other lamps of various sorts were added to the light department, and its work increased. Here we have only to deal with the question from the nursing point of view and to describe the organisation of the nurses' work. The first two nurses were sent to Copenhagen to learn the system in the Finsen Institute itself; one of these nurses was made "sister" of the light department at the London, and on her and on Dr. Sequeira devolved the training of subsequent nurses. The nurses are specially selected, and are sent to the light department for three months; sometimes nurses from other hospitals are taken for training, but this is very rare, and done merely to oblige other institutions. The present sister of the department—Miss Blandford—has a copy of Dr. Sequeira's article in the now defunct *Physician and Surgeon*, which she gives the new probationer to read, and which forms the theoretical introduction to the work, and the practical work is begun on the easiest cases. There are from 100 to 150 patients treated daily at the London, so the routine has to be very carefully adhered to. Each patient is photographed on commencement of the treatment, and the history of the case is taken; arrangements are, where necessary, made for lodgings in the neighbourhood, and when the patient is found insufficiently nourished the sister can, by private charity, generally arrange help in that direction. Each patient attends daily for one hour if treated by the big Finsen lamps. The patient goes into the dressing-room and removes wraps, and takes her towel from the shelf which corresponds with the number she is given; she then sees the doctor or sister in attendance, and the part to be treated is cleaned with an antiseptic (generally a bit of cotton wool dipped in boracic) and the exact part for treatment is marked round with a dermatographic pencil—a blue pencil which marks easily on the skin. The patient then mounts her couch, spreads her towel over the pillows before putting her head down, and having got comfortably into position the nurse adjusts the pressure lens and the treatment begins. You will find in all medical books the phrase that the treatment is "painless," and from a scientific point of view perhaps it is; but the position is constraining, even slight pressure on such parts as the cartilage of the nose for instance, gives pain, and the subsequent dressing and the inflammatory action of the light on the skin causes a certain amount of suffering. So nurses must remember to be gentle and sympathetic. Each séance lasts an hour with the large lamp, and then the patient gets down from the couch, takes her towel with her to the dressing-room, and there the face is dressed again and the patient goes home. Boracic is the favourite dressing, but others are sometimes used, some cases responding marvellously to iodoform. The mark of the pencil is removed by "sesame" oil—an oil got from the seeds of sunflowers. The inflammatory action on the skin is such that redness, swelling, and small blisters appear 12 to 24 hours afterwards, and sometimes these call for a soothing dressing. The treatment goes on for months in most cases, the average time being four months, and before the patient leaves another photograph is taken to compare with the first one. One of the great advantages of light treatment, is not only that it cures, but that it leaves so little scarring and the skin is smooth and flat and pale when healed.

Lupus vulgaris is a tubercular disease of the skin of the face, most often seen in young persons of a consumptive or scrofulous tendency. It is a very hideous and terrible disease, eating away sometimes the whole of the nose and the upper lip, and yet it affects the general health but slightly and does not kill, so that cases go maimed for years. Koch's fluid was a supposed specific, but failed, and though cases have been cured, sometimes very suddenly and very remarkably, it has always been regarded as one of the most intractable maladies. Of course disinfection and care to prevent contagion are strong nursing points. The nurses should wear overalls with short sleeves, each nurse should have her own hand-basin, soap and towel, and should carefully cleanse and disinfect her hands and forearms between each case. The charge nurse should herself disinfect each pressure glass between the cases. The nurses should also wear smoked spectacles to protect their eyes In some cases it is well for the patients to wear spectacles, or the nurse may find it well to cover the patient's eyes with a piece of damp lint and then a circular bit of brown paper to make darkness and coolness. It is only after time and the care of many cases that nurses find out all that there is to learn with regard to the many different points in the treatment. When the mucous membrane of the nose and mouth are affected the ordinary pressure glass cannot be used; indeed, these parts are generally treated by *x*-rays until healing commences, and then a special small conical lens can be used in some cases with the Finsen light. It is in all these details of treatment that the nurse gets trained at the London, and then she is frequently sent to other hospitals to inaugurate or carry on the work there. It is a great work, for Dr. Dawson Turner, in the third edition of his "Practical Medical Electricity," says: "It must now be admitted that in the Finsen light we have a specific for lupus vulgaris. So long ago as September 1899 Dr. Finsen had treated 350 cases of this disease with a satisfactory result in 345."

The original Finsen lamp, however, has great drawbacks; it is very costly to install, and its upkeep is expensive. A lengthy exposure is needed, and the intensity of the rays is diminished by the patient's distance from the light. Naturally attempts have been made to better the lamp, and in a third paper some newer lamps and newer methods will be described, and the latest results given.

(*To be continued.*)

### Queen Alexandra's Imperial Military Nursing Service.

APPOINTMENTS GAZETTED.

THE following is from the *Gazette* of Tuesday evening:—

*Matron-in-Chief.*—Sidney Jane Browne (R R C.) (temporary).

*Principal Matrons.*—Ethel Hope Becher (R R.C.), Caroline Helen Keer (R R.C.).

*Matrons.*—Beatrice Isabel Jones, Florence Ellen Addams-Williams, Mary Cecil Florence Kate Cole (R.R C.), Elizabeth Ferguson, Ann Garriock (R.R C.), Lydia Hardement, Isabella Julia Jerrard (R.R.C.), Marianne Catherine Smith Knox (R.R.C.), Sarah Elizabeth Oram (R.R.C), Gertrude Mary Payne (R.R.C.), May Russell (R R.C.), Gertrude Elizabeth Saunder, Louisa Mary Stewart (R R C), Alice Emily Tait, Martha Thomas (R.R.C.), Sarah Emily Webb (R R.C.), Mary Wilson, Clara Mavesyn Chadwick (R.R C.), Emma Maud McCarthy (R R.C.), Anne Beadsmore Smith (R.R.C.).

*Sisters.* — Blanche Sarah Vaughan, Edith Christine Cheetham (provisionally), Susannah Lamming (provisionally), Gertrude Emily Larner (provisionally), Louisa Mackenzie Lyall (provisionally), Catherine Anderson, Evangelina Beck (provisionally), Edith Body, Alice Sweeting Bond (R R C.), Deborah Violet Briscoe, Ann Cameron, Elizabeth Cox, Emily Agnes Cox, Lucy Matilda Culverwell, Alexina Guthrie, Mary Ethel Harding, Mary Ellen Harper (R R.C.), Mary Grenfell Hill (R R.C.), Isabel Anna Gerrard Kinahan, Harriet McCurdy, Georgina Adeline Magill, Mary Ridley Makepeace, Martha Mark, Ethel Jane Martin, Agnes Annie Murphy, Helen Louisa Neale, Amy Nixon, Elizabeth Treacher Noble (R.R.C.), Hilda Frances Pocock, Wilhelmina Potter, Caroline Hutton Potts, Dora Isabel Rickards, Annie Rose Rose-Innes, Selina Ysibella Snowdon, Clara Kathleen Emily Steel, Lavinia Eliza Caroline Steen, Edith Mary Elizabeth Todd, Lucy Mary Todd, Dorcas Douglas Tripp, Louisa Watson Tulloh (R.R.C.), Mabel Gertrude Ashton Warner, Sarah Lucy Wilshaw (R.R.C.), Janet Walker Wilson, Maria Wright, Henrietta Tarleton Young, Alicia Barker, Francis Mary Hall, Jane Hoadly (R.R.C.), Dorothy Frances Palmer, Margaret Helen McLeish (provisionally), Elizabeth Charlotte Stewart (provisionally), Isabel Graham Willetts (provisionally), Rosabelle Osborne (provisionally).

*Staff Nurses.*—Ethel Julia Marian Keen (provisionally), Mary Louisa Potter (provisionally), Mary Pedler (provisionally), Elizabeth Mabel Bickerdike (provisionally), Ann Fitzgerald (provisionally), Sybil Rosalie Hughes-Hallett (provisionally), Elizabeth Clement Humphreys (provisionally), Margaret Kendall (provisionally), Caroline Conroy Richards Moor (provisionally), Ethel Mary Pettle (provisionally), Laura Annette Rideout (provisionally), Florence Emia Cradoc Watson (provisionally), Agnes Alexander Wilson (provisionally), Maud Mary Blakely (provisionally), Jane Ann Evans, Ada Ruth Myring (provisionally), Mabel Mary Tunley (provisionally), Mildred Mary Bond (provisionally), Annie Florence Byers (provisionally), Kate Ward (provisionally).

### Appointments.

**[No charge is made for announcements under this head, and we are always glad to receive, and publish, appointments. But it is essential that in all cases the school of training should be given.]**

BROMSGROVE WORKHOUSE INFIRMARY. — Miss Martha Clements has been appointed charge nurse. She was trained at St. Giles's Infirmary, Camberwell, and has since been assistant nurse at Kettering Union Infirmary.

BUCKHURST HILL COTTAGE HOSPITAL. Miss Julie A. Jacobs has been appointed sister-in-charge. She was trained at the Kent and Canterbury Hospital, and has since acted as Queen's district nurse at Alderley Edge, Cheshire.

CHRISTCHURCH WORKHOUSE INFIRMARY.—Miss Elizabeth Parkman and Miss Cecilia Ada Fuller have been appointed charge nurses. Miss Parkman was trained at Newport Infirmary, and has since been charge nurse at Ipswich Union Infirmary and Islington Infirmary. Miss Fuller was trained at St. George's Infirmary, Fulham, and has since been nurse at Richmond Union Infirmary, Surrey.

DERBYSHIRE ROYAL INFIRMARY.—Miss Edith Bond has been appointed assistant matron. She was trained at the Wolverhampton General Hospital, and has since been sister at the Middlesbrough Hospital and the Grimsby and District Hospital.

HARTLEY WINTNEY INFIRMARY, HANTS.—Miss G. G. Kirby has been appointed superintendent nurse. She was trained at Southwark Infirmary, East Dulwich, and has since been sister of male wards at Aston Union Infirmary, Birmingham. She holds the L.O.S. certificate.

HOME FOR INFIRM C.O.S. PENSIONERS, SOUTH HAMPSTEAD.—Miss Jessie Ridley has been appointed matron. She was trained at the General Infirmary, Stafford, the Central London Ophthalmic Hospital, and as a Queen's nurse under the auspices of the Hammersmith and Fulham District Nursing Association. She has since been sister at the General Infirmary, Macclesfield, and nurse in charge of the Rawson Convalescent Home, Harrogate. She has also done district nursing in East London, Spalding, and elsewhere. For the last four years she has been sister at Chalfont St. Peter Home for Epileptics, and was matron *pro tem.* for some months.

KIMBERLEY HOSPITAL.—Miss Polly Blakeley has been appointed staff nurse. She was trained at St. George's Hospital, London, and has had considerable experience in private nursing.

SOUTHWARK INFIRMARY, EAST DULWICH GROVE.—Miss Isabel Kemp has been appointed night superintendent. She was trained at the General Hospital, Northampton, and has since been charge nurse at the Sutton Hospital, and ward sister at the Southwark Infirmary.

STIRLING DISTRICT ASYLUM, LARBERT, N.B.—Miss Elizabeth Ann Thompson and Miss Ellen Eastman have been appointed assistant matrons. Miss Thompson was trained at the Edinburgh Royal Infirmary and has been engaged in private nursing for some years in connection with a nursing home in Edinburgh. Miss Eastman was trained at the East Dulwich Infirmary and has since been night superintendent at Fulham Infirmary.

WOLVERHAMPTON AND STAFFORDSHIRE GENERAL HOSPITAL.—Miss Charlotte P. Gash has been appointed sister. She was trained at the Warneford Hospital, Leamington, and has since been a member of the private nursing staff attached to Princess Christian's Home at Windsor, and sister at the Cambridge Hospital, Aldershot. She has also nursed in South Africa for two years and a half.

### The Nurses of the National Hospital for the Paralysed and Epileptic.

A CHAT WITH THE LADY SUPERINTENDENT: BY OUR COMMISSIONER.

THERE are features of special interest in the system of nursing at the National Hospital for the Paralysed and Epileptic, and when I visited the handsome building in Queen Square, Bloomsbury, for the purpose of an interview with the lady superintendent I asked Miss Vernet to tell me both the points of resemblance and those of difference as compared with general institutions.

"There is one point," she replied, "which I think should be emphasised. Our sisters are all required to have had three years' training at a general hospital."

"Then you do not make sisters of the nurses who have been trained here?"

"Not unless after their training here they have had general training. In that case we might be able to have them back again. Our staff nurses have also had general training; and while we have the advantage of their general experience they have that of gaining experience in the nursing of mental diseases and in being taught massage and medical electricity. Part of the staff nurses are on day and part on night duty."

"Of course I knew that male nurses are a feature of the hospital, but, apart from them, what is the extent of the staff?"

PROBATIONERS AND STAFF NURSES.

"There are 13 probationers, 16 staff nurses, nine ward sisters, an electrical and out-patient sister, a night sister, assistant matron, and myself. The period of training is now two years. It was altered last year. The probationers come for a month on trial, and are chosen generally for suitability."

"You mean that they must have special characteristics?"

"They certainly require to be particularly strong in physique. The nervous cases are rather trying to live with, and delicate nurses would not be equal to the strain. We find that 23 is a very good age."

"At what hour in the morning do the day nurses come on duty?"

"Seven. They breakfast previously. During the day they have two hours off duty and half an hour for dressing. They are free at 8 in the evening. Of course they have also sufficient time for meals. Once a fortnight they are off duty from 2 until 9.30, and every third Sunday they are entirely off duty. The staff nurses have three weeks' holiday, and the probationers a fortnight."

THE SISTERS.

"And the sisters?"

"They come on duty at eight, but they have two evenings and one morning off each week. Also they have a long afternoon and a short one off alternate Saturdays. Every third Sunday they are off duty entirely, and they have a calendar month's holiday. Both nurses and sisters are allowed, with permission, to sleep out when their evening off precedes their Sunday off. I give late leave rather freely because I think that the nurses in this hospital ought to have plenty of amusement. In fact, I encourage them to go out as much as possible, either with friends or to some entertainment. Frequently the managers are good enough to forward me tickets and then I send a couple of nurses to the theatre."

NIGHT DUTY.

"What are the hours on duty of the night nurses?"

"From 8.45 P.M. to 8.30 A.M. There is one night nurse on duty in each ward, with a supernumerary between the wards, who helps whenever it is necessary. The addition of the supernumerary has proved very useful. The night nurses take their meals in the ward kitchen which adjoins the wards. They are on duty for three months, and have a night off every month. I find that the plan of changing once in three months works better than that of changing each month."

"Is the night work heavy?"

"It can be heavy. But when an extra nurse is wanted at night I always put one on. Sometimes there is practically very little to do, and at others the night nurses can hardly find an opportunity to get their meals. The sister goes to and fro between the ten wards, and is, of course, in charge of them all during the night."

"You have lately provided additional accommodation for your night staff?"

"Yes, and we find that it is a great convenience. The new house accommodates thirteen nurses, and the others are housed at the top of the hospital. The nurses take all their meals in the hospital, but both sisters and nurses have nice sitting-rooms in their own quarters. The sisters have separate bedrooms, but most of the nurses share a room."

"As to salaries," continued Miss Vernet, "the ward sisters receive £30 a year, the staff nurses £20, and the probationers £12 the first year and £16 the second."

"With uniform?"

"Yes, with full indoor uniform. We do not give outdoor uniform, but the nurses are at liberty to wear it or not as they please."

THE TRAINING.

"I conclude that the probationers are trained in ward work as at other hospitals?"

"Exactly. They are also, however, trained in massage and electricity. The lectures are given by the Registrar, and are on Physiology, Medical and Surgical Nursing. They are given on the Wednesday evenings between November and May, except a brief interval at Christmas. The examinations at the end of the term are both written and verbal, and two prizes are awarded to the most proficient. The certificate given at the end of two years mentions that they have learnt massage and electricity and attended lectures."

"After they have had their training here do the general hospitals shorten their period of training?"

"Some hospitals will take them for shorter periods; two have done very well at the Derby Royal Infirmary."

QUALITIES NEEDED.

"When you have vacancies do you get many applications?"

"If I advertise for a probationer I receive perhaps a couple of dozen applications. I also get plenty of applications if I want a staff nurse. They are not all suitable, for we make it a point to maintain the standard of the nurse, and they do not invariably come up to it. We need nurses who are quiet and gentle, and, above all things, tactful. The training in patience here is good, and it is a pleasure to me to know that our nurses, when they go to other hospitals, are well spoken of."

THE MALE STAFF.

"Now about the male nurses, who form a unique feature of your institution."

"We have nine resident male nurses, who are taken on trial between the age of 21 and 30, for a month, and if found suitable, stay for two years. There is also a non-resident masseur, who is on duty every day. The supply of candidates for position on the male staff is in excess of the demand."

"Are the regulations the same as those of the female nurses?"

"Precisely, save that they are not off duty in the morning. Their off-duty time is either in the afternoon or evening, and they have two weeks' holiday the first year and three the second. They work in the wards as nurses under the sister, and in the other wards they do the massage and electrical treatment as required."

"Which wards are their headquarters?"

"The paying men's ward and the epileptic ward. The male nurses wash the patients, change their clothes, and do anything for them. As to the training, it is the same for them as for the female probationers, and they participate in the prizes awarded after the examination."

No Practical Difficulties.

"Do you encounter any practical difficulties in maintaining at the same time a female and male staff?"

"None whatever. Moreover there is not the slightest doubt that male nurses are very necessary in this hospital. They can do many things that a woman cannot do, such as lifting a heavy man, and bathing the men. We are very careful in choosing them, and the result is that they are very highly thought of outside and do well in private nursing after they leave us. The nursing institutions like to have our male nurses."

"Are they in your judgment thoroughly interested in their work?"

"I think that the desire of the men for training is very keen. We have two or three now who volunteered as nurses in the South African War, and have come here for further training, and generally speaking the male probationers read up for the lectures, and are most anxious to acquit themselves satisfactorily at the examination."

"How do you arrange about their quarters?"

"They are lodged in the steward's house, which is quite close to the hospital. The salary is £12 the first year and £20 the second with uniform.

Pupils and Paying Probationers.

"You have also pupils?"

"Yes, of both sexes, who are non-resident, and are trained in massage and electricity. They practise in the hospital what they have learnt during the course of training. We average in the year two male and four lady pupils every six weeks, and we could have as many more if we could receive them. Each pupil has individual lessons, and pays five guineas for the massage, and five guineas for the electrical course. We also occasionally take one paying female probationer."

"For how long?"

"Thirteen weeks. For this she pays a fee of 13 guineas, and she is boarded and lodged as well as taught. The paying probationer helps in the wards and does ordinary work. I sometimes have a trained nurse as paying probationer."

"I conclude that you were trained here?"

"I have been probationer, staff nurse, sister, and day and night sister here. But I also trained at the Middlesex Hospital, where I was afterwards day sister. I found my previous experience here extremely valuable when I became matron nearly four years ago."

The Care of the Patients.

"Are many of the patients able to attend the services in your nice little chapel?"

"A very fair number, and those who can attend are very glad to do so. There are services on Sunday morning at 9 45, and on Wednesday at 5. Some of the patients are wheeled in their chairs and each set is accompanied by their nurse. Prayers are read at 9 P.M. for the nurses in the chapel."

"Do the nurses go out for walks with the patients?"

"Yes, both the male and female nurses take the epileptics out for walks—not more than six at once. Patients who cannot walk, but are able to go out, are taken in a bath-chair. We allow male nurses to take patients to smoke in the out-patients' hall or the garden; and any nurse, male or female, is encouraged to play or sing in the wards. Every ward has a piano."

"The idea, I gather, is to make the lives of the patients as bright as possible?"

"That is the object in view. I may add that we give the nurses as much off duty as possible because the amount of attention which the patients require often makes the work very heavy. But the nursing is exceptionally interesting, and as I have often six or seven applicants waiting to become probationers, I presume that it is not unattractive."

### Prevention of Splint Sores.

### EXAMINATION QUESTIONS FOR NURSES.

The question for May was as follows:—What precautions should you take to avoid splint sores in cases of excised elbow joint and fractured femur?

No Prizes Gained this Month.

I fear this will cause great disappointment. Such a calamity has only once before overtaken us in the past five years. The reason for this decision is, that no competitor seems to have grasped the fact that pressure from a splint can only be removed by so arranging matters that any projecting part shall be placed in a slight hollow, not by adding pads of wool under the projections, this only increases the discomfort tenfold. Try to remember that to be a really good surgical nurse you must have a certain amount of mechanical and engineering knowledge and understand relative forces. Now how should pressure be obviated in the two cases suggested? With an excised elbow the pressure will be felt at the joint itself. Some surgeons like a splint with an opening, in that case matters are easy, but if the appliance is padded uniformly, relief must be gained by a pad above and below the joint so that that part of the limb, which from its projection will first feel pressure, should rest in a slight hollow.

With fractured thighs there are four special places to receive attention; the outside of the knee, the outside and inside of ankle-joint and above the back of the heel.

I say nothing of the heel itself, because tenderness there and the necessary precautions are needed in all cases where the patient lies continally on his back. For the outside of the knee, pads similar to those used for an excised elbow must be placed above and below the joint, with the same object in view. Care must be taken that the oval opening in the splint at the ankle is in the right position, and pieces of lint, spread with some lubricant, must be wrapped round the ankle before the strapping stirrup is applied, and if the patient is very thin, a further effort to prevent soreness may be made by cutting out a disc in the stirrup on each side.

The best answer sent in is that by "Fountain," and the second best by "Sussex," but neither merit prizes. I hope when this question is repeated that we shall see evidence of more care and thought.

CARELESSNESS IN READING THE QUESTION.

Why have some 30 home nurses answered the question most plainly put on May 9th to "Nurses in the Colonies and abroad generally?"

QUESTION FOR JUNE.

If told to apply a linseed poultice, how should you proceed? Describe the process from arranging materials and implements to application to patient.

THE EXAMINER.

RULES.

The competition is open to all. Answers must not exceed 500 words, and must be written on one side of the paper only. The pseudonym, as well as the proper name and address, must be written on the *same* paper, and *not on a separate sheet.* Papers may be sent in for fifteen days only from the day of the publication of the question. All illustrations strictly prohibited. Failure to comply with these rules will disqualify the candidate for competition. Prizes will be awarded for the two best answers. Papers to be sent to "The Editor," with "Examination" written on the left-hand corner of the envelope.

N.B.—The decision of the examiners is final, and no correspondence on the subject can be entertained.

In addition to two prizes honourable mention cards will be warded to those who have sent in exceptionally good papers.

### Everybody's Opinion.

[Correspondence on all subjects is invited, but we cannot in any way be responsible for the opinions expressed by our correspondents. No communication can be entertained if the name and address of the correspondent are not given as a guarantee of good faith, but not necessarily for publication. All correspondents should write on one side of the paper only.]

### ONE-YEAR-TRAINED NURSES.

"M. A." writes: I should like to know why it should be made so difficult for a one-year-trained nurse to find employment. Many nurses who were trained eleven years ago, when a three-years' course was not considered so necessary, now find that though they may be quite competent and well recommended, they are thrust aside for their more fortunate fellow nurses. Many medical men prefer women to undertake their cases who have had no training, and yet if they require a nurse from an institution she must hold a three years' certificate. Is this fair to those really competent though insufficiently trained nurses? Some have passed the age limit for further training.

[In any sphere of life the better-trained persons naturally have the advantage, though this is often hard to those of more experience. It is not usual for doctors to prefer untrained women to undertake their cases.—ED. THE HOSPITAL.]

### VARIATIONS IN FOOD.

"OCTO" writes: As a vigorous old invalid I read THE HOSPITAL regularly, and a recent article on "Variations in Food" with very particular interest. Having been a victim of many malarial attacks, in Africa, Central America, and other places, cholera and dysentery abroad, and influenza at home, I have to be very careful of my diet in order to fully enjoy my life as, I am approaching my eightieth year. I read of laboratory experiments on food values, and that beef-tea and meat extracts are of no value. If they are not, then the meats of which my beef-tea and mutton-broth are made should contain as much nourishment as they did before these drinks were made; but my dogs and cats will not eat those meats and I don't like to ask my servants to do so! Now I often work or worry very much, and I can't sleep, and I feel ill and weak, and then I get up and go for some meat extracts (I won't name which, they are various) and beef-tea or mutton broth, and I feel comfortable, then sleep soundly and wake up feeling refreshed, strong, and well-nourished! Of course it can't be the nourishment in the beef-tea or meat extracts because the scientists say they don't contain any! When I don't have much mental work or worry, and I take plenty of exercise, I can eat and thrive on bread-and-butter, toast, rice, sago, hominy in winter time, and Spratt's wholemeal biscuits, with cold water to drink, or very weak tea or very weak coffee. Baked potatoes, stewed fruit, come in as luxuries. Dried fish, highly smoked bacon, and even preserved salmon (tinned salmon) I find very suitable occasionally, but the good, scientific, highly recommended, medically correct articles of food, such as milk, milk pudding, eggs, etc., are most detrimental to my health and temper. They send my spirits down to zero, mental depression comes on, and life is not worth living. Sugar does not disagree, but I can't stand milk in my tea or coffee. When at home I can manage my dieting all right; it is when visiting I meet my enemies—eggs, milk, meat, cheese, and pastry. Surely some nurses ought to be able to give us their experience of feeding old people, and whether the human stomach agrees with the laboratory test tubes always or not about beef-tea, etc.

### Artistic Novelties in Bond Street.

BY OUR SHOPPING CORRESPONDENT.

THERE are always pretty things to be seen at the showrooms of Messrs. Jon. Harris & Sons, Limited, 25 Old Bond Street, and just now they are exhibiting a peculiarly fascinating "early tea tray." This is in the shape of a shamrock leaf, with a green and white wickerwork border, the white linen mat and tiny serviette being embroidered with a shamrock to match. Dainty white china completes the whole, and I cannot imagine a more acceptable present to give to anyone who is fortunate enough to be able to indulge in that "early cup' in bed. There is also a smaller tray for an invalid's beef-tea, etc., which is equally pretty. Nurses going on their holidays should certainly be presented, if photography is their chosen hobby, with a snapshot album covered in art linen; while the cyclist will be equally delighted with a road-book in which to enter her feats on the wheel. All the designs shown may be had traced for working in flax, and needlewomen, anxious for new ideas, cannot do better than go and see the great variety of patterns. The dress materials of strong art linen, as well as the embroidery designs, are made at the Derwent Mills, Cockermouth.

### Presentations.

WARNEFORD ASYLUM, OXFORD.—Miss Agnes Thomson, who is leaving Warneford Asylum, Oxford, in order to take up duty as matron of the Borough Asylum, Canterbury, has been presented by the medical superintendent and his wife with a handsome jewel case, by the matron with an amethyst brooch set with pearls, and by the lady patients with many useful gifts as a mark of esteem. Miss Thomson was trained at the Royal Infirmary, Edinburgh, and Glasgow Maternity Hospital. She has since been Queen's district nurse, sister at the Royal Infirmary, Aberdeen, for five years, and matron of James Murray's Royal Asylum for four years. Miss Thomson, who holds the Medico-Psychological certificate, has been at the Warneford Asylum for two years and a half as L.C. and general assistant.

### Echoes from the Outside World.

### Movements of Royalty.

THE King and Queen have been especially interesting themselves of late in matters of Art. Mr. Essenhigh Corke had the honour of sending his water-colour drawings of Knole, Sevenoaks, to Buckingham Palace for the King's inspection on Monday, and on the same day Commandatore Pietro Canonica submitted some photographs of his Works of Art. On Sunday a presentation was made by the British Ambassador in Berlin, Sir Frank Lascelles, on behalf of the King to the 1st Regiment of Prussian Dragoon Guards, of which his Majesty is honorary Colonel. The presentation took the form of a portrait, the work of Herr Emil Fuchs, representing the King in the uniform of the 1st Prussian Dragoon Guards, and wearing the ribbon of the Order of the Black Eagle. Princess Victoria returned to Buckingham Palace on Monday upon the conclusion of her visit to Prince and Princess Charles of Denmark, but did not accompany the King and Queen when they attended the performance of "Rigoletto" at the Royal Opera in the evening.

### Home Arts Exhibition.

QUEEN ALEXANDRA has always shown much interest in the Home Arts and Industries Association, and as on previous occasions she was again a visitor this year to the Exhibition held in the Royal Albert Hall. Amongst the exhibits was a screen, of which the two panels had been embroidered by the Princess of Wales in many delicate hues, and for the second year, Princess Mary of Wales contributed some work of her own, a pair of grey woollen mittens, most surprisingly even and well knitted considering that the maker has as yet only seen six summers. The Queen made several purchases, including a length of heather mixture made by the women who work under the direction of Lady Radnor at Downton, near Longford Castle, and some dainty smocked frocks and flat Puritan bonnets in prettily striped cotton made at Haslemere. The Queen also bought a good many toys, and four discharged soldiers—who have been taught toy-making under the Meath scheme—were rendered happy, not only by being spoken to by her Majesty but because she became the purchaser of the rug, the bookshelf, and the doll's house furniture upon which she saw them engaged. The Windermere handweavers received an order for a dress length of the purest white silk.

### A Centenarian Peeress.

TUESDAY was a memorable day in the life of Lady Glentworth. On that morning she attained her 100th birthday. Being still in good health and rejoicing in the possession of all her faculties, she received the congratulations of her numerous relatives at Marham House, about nine miles from Downham Market. She had also the honour of receiving the congratulations of the King and Queen, together with a beautiful bouquet of flowers. Lady Glentworth, who was two years of age when Nelson died, is a granddaughter of "the beautiful Mrs. Villebois," who will live in posterity as the subject of one of the most famous of Gainsborough's portraits. As a girl she came up frequently to London to spend the season, and the coach in which the journey was performed—drawn by four horses—is still preserved. In the year before Queen Victoria ascended the throne Miss Villebois married Viscount Glentworth, and was present at the Coronation, the girlish grace and dignified bearing of the young Sovereign making a distinct impression upon her. Lady Glentworth still manages all her own business affairs, signs all papers of importance clearly and firmly, and takes the keenest interest in political and other movements, which she is fond of discussing. She is the only centenarian in the peerage.

### Motor-Car Racing.

THE race between motor cars from Paris to Madrid commenced on Sunday, but it led to so many fatalities that the continuance of the racing has been prohibited. The result of the first day's accidents is six deaths, which are likely to be followed by more. A woman was run over and killed; Mr. Porter's car overturned and took fire, he was badly burnt and his companion killed; Mr. Barrow's mechanic was killed, and he himself had his pelvis and one of his thighs fractured, and his leg may have to be amputated; a car driven by M. Rénault was thrown into a ditch, and he succumbed to his injuries. At one time this car was reported to have attained a speed of 88 miles an hour. Madame du Gast, the lady motorist, drove her own car, and reached Bordeaux in safety. Even in England, at the Bristol Post Office sports on Saturday, a lamentable disaster occurred. Three motor cycles were racing when two collided, and both men and machines were thrown amongst the spectators. Two have since died, and a third is in a critical state.

### Sale of Pictures.

VERY high prices for pictures were realised during the past week. At an auction in King Street Mr. Martin Colnaghi paid 14,000 guineas for a picture of "Sir John Sinclair of Ulbster" painted by Raeburn. The works of this Scottish painter have of late risen very rapidly in public favour. Twenty-five years ago 49 of his works only realised £6,000. Last year four alone produced £14,910. On Saturday at Christie's auction rooms the collection of pictures made by Mr. Reginald Vaile and other smaller collections were brought under the hammer. Four pictures by Boucher, "The Fortune Teller," "The Love Message," "Love's Offering," and "Evening" were sold for 22,300 guineas; "Venus and Mars," by Paul Veronese, for £6,000, and Nallier's portrait of "The Comtesse de Neubourg and her Daughter" for £4,500. "The Portrait of a Young Lady," by Gainsborough, had a romance of its own. A lady from Worthing came up to London a short time since with a picture sadly dirty and neglected. She was offered a couple of pounds for it by a Bond Street House, but refused the munificent offer. On Saturday it was sold for £9,000.

### Death of Max O'Rell.

THE author of "John Bull and his Island" died in Paris on Sunday evening. M. Paul Blouet was widely known by his pseudonym of "Max O'Rell." He commenced his career as a cavalry officer in the French Army in 1867, was made a prisoner at Sedan, and was severely wounded during the Commune. In 1872 he came to England as the correspondent of French papers, and in 1876 settled down as French master at St. Paul's School. This position he resigned in 1884 owing to the fame and money which he earned by the publication of "John Bull and his Island." His subsequent works, some of which have also been extremely popular, included "John Bull's Daughter," "The Dear Neighbours," "Drat the Boys," "John Bull & Co.," and "Her Royal Highness Woman." Even more remarkable than the success achieved by Max O'Rell in literature was the result of his appearance on the lecture platform. In 1891 he went on a lecturing tour round the world, and delivered 446 lectures.

### Popular Passenger Steamers.

ON Saturday next, May 30, the popular passenger steamers, *Royal Sovereign* and *Koh-i-Noor*, belonging to the New Palace Steamers, Limited, will commence running, for the summer season, from London Bridge (Old Swan Pier) to Southend, Margate, and Ramsgate, at the same times of sailing as last year. *Koh-i-Noor* leaves for Southend and Margate daily at 8.20, and *Royal Sovereign* at 9.20 for Margate and Ramsgate. Reduced fares will be conceded to parties of 12 and upwards.

### For Reading to the Sick.

WHITSUNTIDE.

Holy Ghost! my Comforter! .
Now from highest Heaven appear,
Shed Thy gracious radiance here.

Thou the heart's most precious Guest,
Thou of Comforters the best,
Give to us, Thy people, rest.

Bend the stubborn will to Thine,
Melt the cold with fire Divine,
Erring hearts aright incline.
*17th Century Hymn.*

Oh! blessed news, that God Himself is the Comforter! Blessed news, that He who strikes will also heal; that He who gives the cup of sorrow will also give the strength to drink it! Blessed news, that chastisement is not punishment, but the education of a Father! Blessed news, that our Comforter is the Spirit who comforted Christ the Son Himself; who proceeds both from the Father and the Son, and will tell us that in Christ we are really and literally the children of God, who may cry to Him in our extreme need, "Father," with full understanding of all that royal word contains!—*Charles Kingsley.*

Jesus Christ declared that the Spirit should come upon men as an abiding possession for all time. "I will pray the Father, and He shall give you another Comforter, that He may be with you for ever, even the Spirit of Truth."

If we would know the secret of heavenly-mindedness, of Christ-like uprightness, purity, gentleness, truthfulness, self-sacrifice, and charity, we must confess the sanctifying agency of the Holy Spirit, uniting the souls of men to Jesus Christ, and enabling them to follow His perfect example. It is only when men yield to, and co-operate with, the blessed influences of the Spirit of God, that others will "take knowledge of them, that they have been with Jesus."

Grace is much more than the goodwill or favour of God towards mankind. It is rather of the nature of actual power, or spiritual force, bestowed upon us, and working in us. Grace is a spiritual and supernatural gift of God, by which we are rendered acceptable to Him, and empowered to serve Him truly. Grace is the life of our Incarnate Lord within the soul. It is worked in us by the Holy Spirit, who is the Life-Giver—the Giver of Christ, who is the Life.—*Vernon Staley.*

Blest Spirit, I would yield myself to Thee,
Do more for me than I can ask or think;
Let me Thy holy habitation be
And daily deeper from Thy fullness drink.
*C. Forsyth.*

Peace is God's direct assurance
To the souls that win release
From this world of hard endurance—
Peace—He tells us—only Peace!
*Houghton.*

### Notes and Queries.

**REGULATIONS.**

The Editor is always willing to answer in this column, without any fee, all reasonable questions, as soon as possible.

But the following rules must be carefully observed:—

1. Every communication must be accompanied by the name and address of the writer.
2. The question must always bear upon nursing, directly or indirectly.

If an answer is required by letter a fee of half-a-crown must be enclosed with the note containing the inquiry. We cannot undertake to forward letters addressed to correspondents making inquiries unless a stamped envelope is enclosed.

*Massage and Midwifery.*

(78) 1. Can you tell me where I can learn massage and electricity for a small fee? 2. Can a certificated midwife take up monthly nursing when out of cases? 3. Do you consider six months' training enough to make a midwife thorough in all duties?—*Anxious Inquirer.*

1. Apply to the National Hospital for the Paralysed and Epileptic, Queen's Square, Bloomsbury, W.C. 2. Yes. 3. It depends upon the intelligence and aptitude of the pupil.

*"Blackie's Physiology."*

(79) Will you kindly tell me the full title of the book "Blackie's Physiology," and by whom it is published?—*E. M. H.*

The work you refer to is "Animal Physiology for Beginners," by Vincent T. Murché, published by Blackie and Son, price 1s. 4d. net cash, post free. You can obtain it from Young J. Pentland, publisher, 38 West Smithfield, London, E.C.

*Hospital Training.*

(80) I am desirous of getting a three years' training in a general hospital which will fit me for Queen Alexandra's Nursing Service. As cost is a consideration with me will you kindly give me the names of institutions where probationers are trained and receive a small salary the second and third year?—*E. L. H.*

Consult the "Nursing Profession: How and Where to Train."

Will you kindly tell me if the West Suffolk Hospital, Bury St. Edmunds, is a recognised training school for probationers, also how many beds there are?—*E. H.*

The West Suffolk Hospital has 62 beds, of which the number occupied averages 45. A good course of training is provided, but you had better write to the Secretary, The Local Government Board, Whitehall, S.W., and ask if it is recognised as qualifying for the post of superintendent nurse before you enter on your training.

*Cleansing*

(81) 1. Will you kindly tell me how I can clean a breast exhauster, also the feet of elastic stockings. 2. Is Southend-on-Sea a good health resort for a rheumatic patient, and if warm sea baths can be obtained there?—*N. M.*

Wash well in soap and water, using swabs of cotton wool and a soft brush to remove all encrustations; then plunge the exhauster into cold water to which a suitable antiseptic has been added until it is wanted for use again. If the exhauster is well washed, immediately after use, with soap and water, and either plunged into cold water, or exposed to the fresh air, it will keep sweet. Elastic stockings can be washed if cold water is used, and they are dried carefully in a cool place out of the sun. 2. Consult your medical man as to the suitability of Southend for your patient.

*Theory.*

(82) Are nurses considered qualified to receive certificates when they have not been taught theory?—*Perplexed.*

Any institution can give a certificate, but unless those holding it have been properly taught it is of no use. The institution you mention is not recognised as a training school for nurses.

**Important Nursing Textbooks.**

"The Nursing Profession: How and where to Train." 2s. net; 2s. 4d. post free.

"A Handbook for Nurses." (New Edition). 5s. net; 5s. 4d. post free.

"The Human Body." 5s. post free.

"Ophthalmic Nursing." (New Edition). 3s. 6d. net; 3s. 10d. post free.

"Gynæcological Nursing." 1s. post free.

"Art of Feeding the Invalid." (Popular Edition). 1s. 6d. post free.

"Practical Hints on District Nursing." 1s. post free.

## Figures and Tables

**Figure f1:**